# Association of Liver-Directed Local Therapy With Overall Survival in Adults With Metastatic Intrahepatic Cholangiocarcinoma

**DOI:** 10.1001/jamanetworkopen.2019.11154

**Published:** 2019-09-13

**Authors:** Nikhil T. Sebastian, Yubo Tan, Eric D. Miller, Terence M. Williams, Anne M. Noonan, John L. Hays, Sherif Abdel-Misih, Dayssy Alexandra Diaz

**Affiliations:** 1 Department of Radiation Oncology, The Ohio State University Comprehensive Cancer Center–Arthur G. James Cancer Hospital and Richard J. Solove Research Institute, Columbus; 2 Department of Biomedical Informatics, The Ohio State University College of Medicine, Columbus; 3 Division of Medical Oncology, Department of Internal Medicine, The Ohio State University Comprehensive Cancer Center–Arthur G. James Cancer Hospital and Richard J. Solove Research Institute, Columbus; 4 Division of Surgical Oncology, Department of Surgery, The Ohio State University Comprehensive Cancer Center–Arthur G. James Cancer Hospital and Richard J. Solove Research Institute, Columbus

## Abstract

**Importance:**

Intrahepatic cholangiocarcinoma is an aggressive hepatobiliary malignant neoplasm characterized by local progression and frequent metastasis. Definitive local therapy to the liver in the setting of metastatic intrahepatic cholangiocarcinoma may improve overall survival.

**Objective:**

To compare the overall survival of patients with metastatic intrahepatic cholangiocarcinoma treated with chemotherapy alone vs chemotherapy with definitive liver-directed local therapy.

**Design, Setting, and Participants:**

This cohort study used the National Cancer Database to identify 2201 patients with metastatic intrahepatic cholangiocarcinoma diagnosed between January 2004 and December 2014 who received chemotherapy with or without hepatic surgery or external beam radiation to a dose 45 Gy or higher. Multiple imputation, Cox proportional hazards, propensity score matching, and landmark analysis were used to adjust for confounding variables. Analyses were performed between September 2018 and February 2019.

**Exposures:**

Chemotherapy alone and chemotherapy with liver-directed surgery or radiation.

**Main Outcomes and Measures:**

Overall survival.

**Results:**

A total of 2201 patients (1131 [51.4%] male; median [interquartile range] age, 63 [55-71] years) who received chemotherapy alone (2097 [95.3%]) or chemotherapy with liver-directed local therapy (total, 104 [4.7%]; surgery, 76 [73.1%]; radiation, 28 [26.9%]) were identified. Patients treated with chemotherapy alone had larger median (interquartile range) primary tumor size (7.0 [4.4-10.0] cm vs 5.6 [4.0-8.3] cm; *P* = .048) and higher frequency of lung metastases (383 [25.9%] vs 7 [6.7%]; *P* = .004). Patients treated with liver-directed local therapy had higher frequency of distant lymph node metastases (34 [32.7%] vs 528 [25.2%]; *P* = .045). Liver-directed local therapy was associated with higher overall survival compared with chemotherapy alone on multivariable analysis (hazard ratio [HR], 0.60; 95% CI, 0.48-0.74; *P* < .001). A total of 208 patients treated with chemotherapy alone were propensity score matched with 104 patients treated with chemotherapy plus liver-directed local therapy. Liver-directed local therapy continued to be associated with higher overall survival (HR, 0.57; 95% CI, 0.44-0.74; *P* < .001), which persisted on landmark analysis at 3 months (HR, 0.61; 95% CI, 0.47-0.79; log-rank *P* < .001), 6 months (HR, 0.68; 95% CI, 0.50-0.92; log-rank *P* = .01), and 12 months (HR, 0.68; 95% CI, 0.47-0.98; log-rank *P* = .04).

**Conclusions and Relevance:**

In this study, the addition of hepatic surgery or irradiation to chemotherapy was associated with higher overall survival when compared with chemotherapy alone in patients with metastatic intrahepatic cholangiocarcinoma. These findings may be valuable given the paucity of available data for this disease and should be validated in an independent cohort or prospective study.

## Key Points

**Question:**

Is the addition of liver-directed local therapy to chemotherapy associated with an improvement to overall survival for patients with metastatic intrahepatic cholangiocarcinoma?

**Findings:**

This cohort study, using data from 2201 patients with metastatic intrahepatic cholangiocarcinoma from the National Cancer Database, found statistically significant higher overall survival in patients treated with chemotherapy and liver-directed surgery or irradiation vs patients treated with chemotherapy alone.

**Meaning:**

The findings suggest that patients with metastatic intrahepatic cholangiocarcinoma who receive chemotherapy, definitive treatment to the primary liver tumor in the form of surgery or radiation is associated with improved overall survival.

## Teaser

This cohort study compares the overall survival of patients with metastatic intrahepatic cholangiocarcinoma treated with chemotherapy alone vs chemotherapy with definitive liver-directed local therapy.

## Introduction

Cholangiocarcinoma is the most common biliary malignant neoplasm and second most common primary hepatic malignant neoplasm in the United States, and its incidence continues to increase globally.^1,2^ Primary therapy for localized intrahepatic cholangiocarcinoma (ICC), which is anatomically and biologically distinct from extrahepatic perihilar and distal cholangiocarcinomas, entails hepatic resection with or without adjuvant chemotherapy and hepatic irradiation. For advanced or metastatic disease, systemic therapy with consideration of locoregional therapy is the standard of care. Particularly for advanced and metastatic disease, prognosis is dismal, typified by a median overall survival of 12 months.^3,4^

Recurrent cholangitis and liver abscess may contribute to morbidity and mortality in patients with advanced disease.^5^ Despite this, the role of local therapy in the metastatic setting is poorly defined. Given the rarity of ICC, observational studies leveraging national databases are particularly beneficial for evaluating patterns of care and survival. To this end, we sought to evaluate the overall survival of patients with metastatic ICC treated with chemotherapy with local treatment to the liver, in the form of hepatic resection or definitive irradiation, using the National Cancer Database (NCDB), a nationwide hospital-based registry encompassing 70% of newly diagnosed malignant neoplasms in the United States.

## Methods

### Study Population

**Figure 1.**
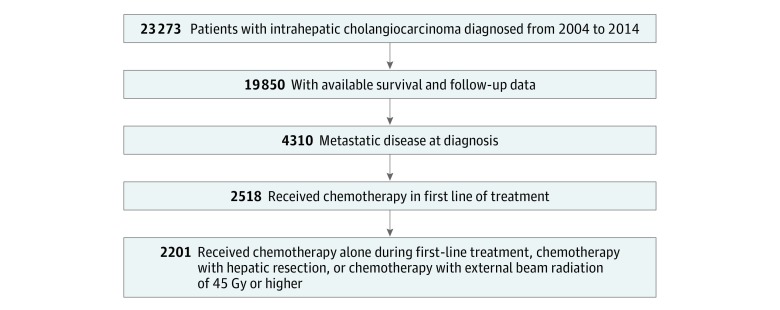
Patient Selection Schema for the Analyzed Cohort All analyzed patients had distant metastatic (M1) disease at the time of diagnosis, per the American Joint Committee on Cancer.^6^ All patients had chemotherapy as part of their first line of treatment, defined as all methods of treatment recorded in the treatment plan and administered to the patient before disease progression or recurrence.

Figure 1 shows the patient selection schema for the analyzed cohort.^6^ We identified patients diagnosed with ICC between January 2004 and December 2014 with available follow-up and survival data. We selected only patients with metastatic disease (M1, per the American Joint Committee on Cancer^6^) who received chemotherapy in the first line of treatment, defined as all methods of treatment recorded in the treatment plan and administered to the patient before disease progression or recurrence. Of these, we analyzed only those patients who received chemotherapy alone or with hepatic resection or external beam radiation therapy to a total dose of 45 Gy or higher. Data analysis took place from September 2018 to February 2019. The Ohio State University institutional review board approved this study as exempt, with a waiver of informed consent because of the absence of risk to study participants. The data available were deidentified, and the analysis followed the Strengthening the Reporting of Observational Studies in Epidemiology (STROBE) reporting guideline.^7^

### Statistical Analysis

Survival was calculated from the time of diagnosis. Cox multivariable regression was performed on the basis of age, sex, race, Charlson Comorbidity Index score, primary tumor focality, primary tumor vascular invasion, T stage, N stage, tumor size, year of diagnosis, insurance status, treatment facility type, and presence of bone, lung, and/or distant lymph node metastasis. For Cox multivariable regression, missing values for all categorical values were handled as a unique level. For sensitivity analysis, missing data were multiply imputed using chained equations to generate 10 imputed data sets^8^ using the covariates from the regression analysis as well as education status, residential population, hospital distance, and the survival outcome.^9^ Using the multiply imputed data, Cox models were repeated and hazard ratio (HR) estimates were pooled using Rubin rules.^8^ Additionally, 2:1 nearest-neighbor propensity score matching (caliper, 0.1), using the same covariates used in the multivariable regression, was performed on each imputed data set and pooled.^10^ Median follow-up times for all patients and living patients were calculated, and overall survival of the matched cohorts was assessed with Kaplan-Meier analysis. To account for immortal time bias,^11^ sequential landmark analysis at 3 months, 6 months, and 1 year was performed on the matched data set.

All statistical analyses were performed with R version 3.5.1 (R Project for Statistical Computing). All *P* values were 2-sided and considered statistically significant if less than .05.

## Results

Our final cohort consisted of 2201 patients (1131 [51.4%] male; median [interquartile range (IQR)] age, 63 [55-71] years) who received chemotherapy either alone (2097 [95.3%]) or with liver-directed local therapy (LDLT) (104 [4.7%]). The local treatment in the LDLT cohort consisted of either hepatic surgery (76 [73.1%]) or irradiation (28 [26.9%]). In the patients who received liver-directed surgery, surgery type was segmental or wedge resection (36 [47.4%]), lobectomy (25 [32.9%]), partial lobectomy (7 [9.2%]), or not otherwise specified (8 [10.5%]). In the group that received liver-directed radiation, the median dose was 50.4 Gy (range, 45-72 Gy), and the median number of fractions was 25 (range, 4-40). Median (IQR) follow-up for both groups was 8.1 (3.8-15.6) months. Median (IQR) follow-up for living patients was 20.1 (7.4-35.9) months. Table 1 lists the patient characteristics of the cohort. The groups were well balanced for most variables. There was a higher percentage of patients with lung metastases in the chemotherapy alone cohort (383 [25.9%] vs 7 [6.7%]; *P* = .004), and patients had larger tumor size (median [IQR], 7.0 [4.4-10.0] cm vs 5.6 [4.0-8.3] cm; *P* = .048). Patients in the cohort that received LDLT had a higher proportion of distant lymph node metastasis (34 [32.7%] vs 528 [25.2%]; *P* = .045).

**Table 1.** Patient and Disease Characteristics of the Analyzed Cohort, Stratified by Receipt of LDLT

Characteristic	No. (%)		*P* Value
	Chemotherapy Alone (n = 2097)	Chemotherapy With LDLT (n = 104)	
Age, median (IQR), y	63 (55-71)	61.5 (54-70)	.32^a^
Sex			
Male	1085 (51.7)	46 (44.2)	.16^b^
Female	1012 (48.3)	58 (55.8)	
Race			
White	1765 (84.2)	89 (85.6)	.96^b^
Black	181 (8.6)	9 (8.7)	
Other^c^	123 (5.9)	5 (4.8)	
Unknown	28 (1.3)	1 (1.0)	
Charlson Comorbidity Index score			
0	1504 (71.7)	73 (70.2)	.50^b^
1	416 (19.8)	25 (24.0)	
2	108 (5.2)	5 (4.8)	
≥3	69 (3.3)	1 (1.0)	
Insurance status			
No insurance	84 (4.0)	5 (4.8)	.26^b^
Private	900 (42.9)	48 (46.2)	
Government	1063 (50.7)	46 (44.2)	
Unknown	50 (2.4)	5 (4.8)	
Treatment facility			
Community cancer program	130 (6.2)	5 (4.8)	.67^b^
Comprehensive community cancer program	723 (34.5)	30 (28.8)	
Academic or research institution	983 (46.9)	55 (52.9)	
Integrated network cancer program	198 (9.4)	10 (9.6)	
Unknown	63 (3.0)	4 (3.8)	
Year of diagnosis, median (IQR)	2011 (2009-2013)	2011 (2009- 2013)	.28^**a**^
T stage^d^			
1	224 (10.7)	12 (11.5)	.12^b^
2	586 (27.9)	24 (23.1)	
3	350 (16.7)	28 (26.9)	
4	182 (8.7)	8 (7.7)	
Unknown	755 (36.0)	32 (30.8)	
Primary tumor size, median (IQR), cm^e^	7.0 (4.4-10.0)	5.6 (4.0-8.3)	.048^a^
Primary tumor focality			
Unifocal	340 (16.2)	20 (19.2)	.52^b^
Multifocal	616 (29.4)	26 (25.0)	
Unknown	1141 (54.4)	58 (55.8)	
N stage^d^			
0	689 (32.9)	32 (30.8)	.78^b^
1	863 (41.2)	42 (40.4)	
Unknown	545 (26.0)	30 (32.8)	
Bone metastasis			
No	1262 (60.2)	62 (59.6)	.70^b^
Yes	188 (9.0)	7 (6.7)	
Unknown	647 (30.9)	35 (33.7)	
Lung metastasis			
No	1055 (50.3)	62 (59.6)	.004^b^
Yes	383 (18.3)	7 (6.7)	
Unknown	659 (31.4)	35 (33.7)	
Distant lymph node metastasis			
No	1223 (58.3)	48 (46.2)	.045^c^
Yes	528 (25.2)	34 (32.7)	
Unknown	346 (16.5)	22 (21.2)	

Abbreviations: IQR, interquartile range; LDLT, liver-directed local therapy.

^a^ Determined with Mann-Whitney *U* (Wilcoxon rank sum) test.

^b^ Determined with Fisher exact test.

^c^ Includes American Indian, Asian, and Native Hawaiian or Pacific Islander.

^d^ Classification per the American Joint Committee on Cancer, 7th edition.

^e^ Patients with available data: chemotherapy alone, 931; chemotherapy with LDLT, 66.

**Figure 2.**
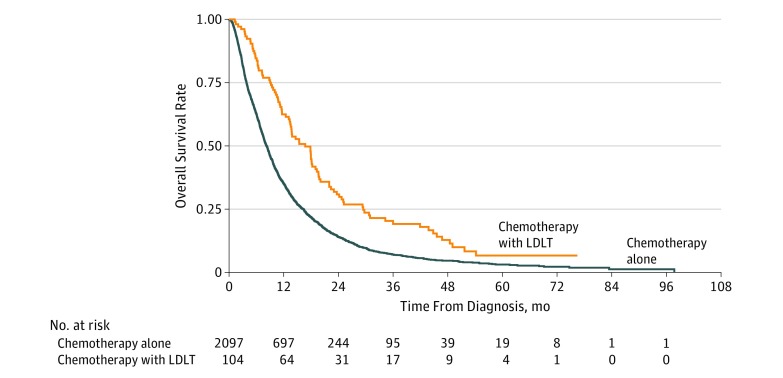
Kaplan-Meier Survival Curves for Overall Survival of Patients Treated With and Without Liver-Directed Local Therapy (LDLT) Log-rank test for overall survival in unmatched cohorts, *P* < .001.

Median overall survival was 8.3 (95% CI, 7.8-8.7) months for the chemotherapy alone cohort and 16.7 (95% CI, 13.4-19.6) months for the LDLT cohort (Figure 2). Univariate analysis found that LDLT was associated with improved overall survival (HR, 0.57; 95% CI, 0.46-0.71; *P* < .001). On multivariable Cox regression, LDLT was again associated with improved overall survival (adjusted HR, 0.60; 95% CI, 0.45-0.79; *P* < .001). There was no statistically significant difference in survival between patients in the LDLT group treated with hepatic irradiation vs surgical resection (HR 1.37; 95% CI, 0.76-2.46; *P* = .30). Other factors associated with survival on multivariable analysis, regardless of adjuvant therapy, included female sex (HR, 0.85; 95% CI, 0.74-0.98; *P* = .03) and treatment at an academic facility (HR, 0.73; 95% CI, 0.53-1.00; *P* = .047). Factors associated with lower survival included Charlson Comorbidity Index score of 1 (HR, 1.23; 95% CI, 1.04-1.47; *P* = .02) and 3 or greater (HR, 1.64; 95% CI, 1.03-2.60; *P* = .04), presence of bone metastases (HR, 1.67; 95% CI, 1.29-2.16; *P* < .001), and presence of lung metastases (HR, 1.45; 95% CI, 1.20-1.77; *P* < .001).

**Figure 3.**
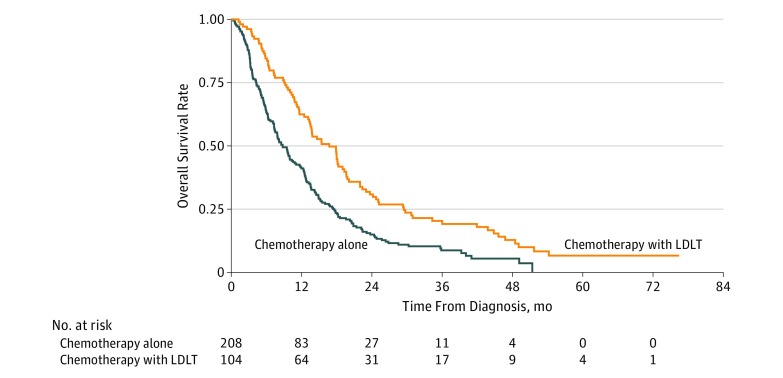
Kaplan-Meier Survival Curves for Overall Survival of Propensity Score Matched Patients Treated With or Without Liver-Directed Local Therapy (LDLT) Log-rank test for overall survival in propensity score matched cohorts, *P* < .001.

After multiple imputation, LDLT remained associated with improved overall survival using multivariable Cox regression on the imputed data set (HR, 0.60; 95% CI, 0.48-0.74; *P* < .001) (Table 2). Additional factors associated with mortality included age, Charlson Comorbidity Index score (score of 1: HR, 1.23; 95% CI, 1.10-1.38; *P* < .001; score of ≥3: HR, 1.49; 95% CI, 1.15-1.92; *P* = .002), treatment at an academic or research facility (HR, 0.80; 95% CI, 0.65-0.98; *P* = .03), early year of diagnosis (HR, 0.98; 95% CI, 0.96-0.996; *P* = .02), presence of bone metastases (HR, 1.30; 95% CI, 1.03-1.65; *P* = .02), and presence of lung metastases (HR, 1.24; 95% CI, 1.07-1.44; *P* = .004). After propensity score matching of the imputed data set, there were 208 patients in the chemotherapy-alone cohort and 104 patients in the LDLT cohort (eTable in the Supplement). Standardized mean differences for all covariates were less than 0.1. Compared with chemotherapy alone, LDLT continued to be associated with improved overall survival (HR, 0.57; 95% CI, 0.44-0.74; *P* < .001) (Figure 3).

**Table 2.** Multivariable Cox Proportional Hazards for Overall Mortality Using the Multiply Imputed Data Set

Variable	HR (95% CI)	*P* Value
Treatment		
Chemotherapy alone	1 [Reference]	NA
Chemotherapy with LDLT	0.60 (0.48-0.74)	<.001
Age	1.01 (1.00-1.01)	.003
Sex		
Male	1 [Reference]	NA
Female	0.86 (0.79-0.95)	.002
Race		
White	1 [Reference]	NA
Black	1.05 (0.89-1.24)	.54
Other	1.00 (0.82-1.23)	.98
Charlson Comorbidity Index score		
0	1 [Reference]	NA
1	1.23 (1.10-1.38)	<.001
2	1.29 (1.05-1.59)	.014
≥3	1.49 (1.15-1.92)	.002
Insurance status		
No insurance	1 [Reference]	NA
Private	0.81 (0.63-1.03)	.09
Government	0.84 (0.65-1.08)	.17
Treatment facility		
Community cancer program	1 [Reference]	NA
Comprehensive community cancer program	1.01 (0.82-1.25)	.48
Academic or research institution	0.80 (0.65-0.98)	.03
Integrated network cancer program	0.92 (0.72-1.16)	.47
Year of diagnosis	0.98 (0.96-0.996)	.02
T stage^a^		
1	1 [Reference]	NA
2	1.09 (0.85-1.39)	.48
3	1.14 (0.86-1.52)	.35
4	1.21 (0.88-1.68)	.22
Tumor focality		
Unifocal	1 [Reference]	NA
Multifocal	1.01 (0.83-1.22)	.93
N stage^a^		
0	1 [Reference]	NA
1	1.06 (0.95-1.17)	.28
Bone metastasis		
No	1 [Reference]	NA
Yes	1.30 (1.03-1.65)	.02
Lung metastasis		
No	1 [Reference]	NA
Yes	1.24 (1.07-1.44)	.004
Distant lymph node metastasis		
No	1 [Reference]	NA
Yes	0.90 (0.79-1.03)	.13

Abbreviations: HR, hazard ratio; LDLT, liver-directed local therapy; NA, not applicable.

^a^ Classification per the American Joint Committee on Cancer, 7th edition.

Sequential landmark analysis demonstrated statistically significant improvement in overall survival with LDLT for patients who lived longer than 3 months (HR, 0.61; 95% CI, 0.47-0.79; log-rank *P* < .001), 6 months (HR, 0.68; 95% CI, 0.50-0.92; log-rank *P* = .01), and 12 months (HR, 0.68; 95% CI, 0.47-0.98; log-rank *P* = .04) (eFigure in the Supplement). As an additional measure to mitigate selection bias favoring responders, we analyzed 93 patients receiving LDLT (89.4%) with available treatment time data. Median (IQR) time of surgery for 66 patients was 29.5 (15.0-49.8) days prior to chemotherapy. Median (IQR) time to initiation of radiotherapy for 27 patients was 1 (0-54) days after chemotherapy. When comparing 41 patients treated with chemotherapy within 30 days with LDLT with 52 patients treated outside of 30 days, there was no statistically significant difference in survival (HR, 1.14; 95% CI, 0.73-1.76; *P* = .57). When selecting only those patients who received LDLT within 30 days of chemotherapy initiation, LDLT continued to be associated with improved overall survival when compared with chemotherapy alone (HR, 0.67; 95% CI, 0.48-0.93; *P* = .02).

## Discussion

To our knowledge, this is the first study evaluating survival benefit with the addition of primary tumor-directed therapy in the setting of metastatic biliary cancer. We found that hepatic resection or definitive irradiation to a dose 45 Gy or higher was associated with improved overall survival in metastatic ICC. Notably, this finding remained after accounting for key clinical confounders, such as presence of bone and lung metastases.

While the traditional paradigm of metastatic cancer is that patients with disseminated disease are not suitable for (and do not derive survival benefit from) definitive or aggressive local therapy, a more nuanced understanding of metastatic disease has emerged in recent years. The identification of an intermediate state between locally advanced and widely metastatic cancer has led to studies suggesting disease-progression and survival benefits with local therapy for oligometastatic disease.^12,13,14,15,16^ This strategy pertains not only to untreated, limited metastases but also to the primary tumor. In the setting of metastatic prostate cancer, retrospective studies suggest overall survival and cancer-specific survival benefit with treatment of the primary tumor.^17,18^ For oligometastatic colon cancer, aggressive treatment of the metastatic and primary tumor is the standard of care.^19,20^ Although the elimination of limited subclones with metastatic potential within the primary tumor is itself an intriguing rationale for local therapy,^21^ the lethality of malignant hepatic failure adds further justification for liver-directed therapy for hepatobiliary malignant neoplasms.^22,23^

The high rate of dissemination and relapse seen in biliary malignant neoplasms has established systemic cytotoxic chemotherapy as the standard of care.^24^ While the propriety of primary-directed therapy in a cohort typified by median overall survival of only 1 year may prompt skepticism, continued improvements in operative and radiotherapeutic management may help to facilitate the integration of primary-directed therapy into the metastatic treatment paradigm by enhancing convenience, cost, and toxicity profile.^25,26,27^ Particularly in the case of radiotherapy, advances in treatment planning have allowed the delivery of ablative doses of radiotherapy in 15 or less treatments for hepatobiliary malignant neoplasms, with minimal toxic effects, even with concurrent cytotoxic chemotherapy.^28^

### Limitations

There are several limitations of this study that should be noted. First, the number of patients in the cohort that received liver-directed local therapy is small, and despite attempts to adjust for numerous confounders, including the presence of metastases in key sites, such as bone and lung, there is likely some selection bias for which we are unable to account. Despite the overall robustness and quality control of the NCDB,^29^ the database lacks key variables that would have been informative for this study. Namely, while we were able to account for the presence of metastases, no data regarding the overall number and volume of metastatic lesions was available. It is possible that patients who received primary-directed treatment were those with more limited systemic disease burden, particularly in the case of patients who received surgery, given the delay in chemotherapy administration that was observed. Additionally, data for a few variables were missing for a significant proportion of patients, and despite the use of multiple imputation, bias may have been introduced.^30^ Further, there was no available data regarding specific chemotherapy agents, disease control end points, or performance status, limitations that are inherent to the NCDB.

## Conclusions

This cohort study found that receipt of liver-directed local therapy, in the form of hepatic resection or external beam irradiation to a dose of 45 Gy of higher, was associated with improved survival in the setting of metastatic ICC. These findings should be validated and investigated more thoroughly in independent cohorts containing data regarding metastatic disease burden (eg, number and size of metastatic lesions), tumor control, and patterns of failure. If validated, these findings might suggest that aggressive local therapy of the primary tumor in the setting of metastatic ICC may benefit appropriately selected subsets of patients.

## Notes

Supplement. **eFigure. **Sequential Landmark Kaplan-Meier Curves, Stratified by Receipt of Liver-Directed Local Therapy (LDLT) **eTable. **Patient and Disease Characteristics of the Propensity Matched Cohort, Stratified by Receipt of Liver-Directed Local Therapy (LDLT)
