# Supplementary material for: Association of Liver-Directed Local Therapy With Overall Survival in Adults With Metastatic Intrahepatic Cholangiocarcinoma
Source: JAMA Netw Open. 2019 Sep 13;2(9):e1911154. doi: 10.1001/jamanetworkopen.2019.11154 (PMC6745054; doi:10.1001/jamanetworkopen.2019.11154)
Supplement: Supplement. — eFigure. Sequential Landmark Kaplan-Meier Curves, Stratified by Receipt of Liver-Directed Local Therapy (LDLT) eTable. Patient and Disease Characteristics of the Propensity Matched Cohort, Stratified by Receipt of Liver-Directed Local Therapy (LDLT) [file jamanetwopen-2-e1911154-s001.pdf]

Supplementary Online Content

Sebastian NT, Tan Y, Miller ED, et al. Association of liver-directed local therapy with overall survival in adults with metastatic intrahepatic cholangiocarcinoma. *JAMA Netw Open*. 2019;2(9):e1911154. doi:10.1001/jamanetworkopen.2019.11154

eFigure. Sequential Landmark Kaplan-Meier Curves, Stratified by Receipt of Liver-Directed Local Therapy (LDLT)

eTable. Patient and Disease Characteristics of the Propensity Matched Cohort, Stratified by Receipt of Liver-Directed Local Therapy (LDLT)

This supplementary material has been provided by the authors to give readers additional information about their work.

© 2019 Sebastian NT et al. *JAMA Network Open.*

eFigure. Sequential Landmark Kaplan-Meier Curves, Stratified by Receipt of Liver-Directed Local Therapy (LDLT)

(A) 3-Months

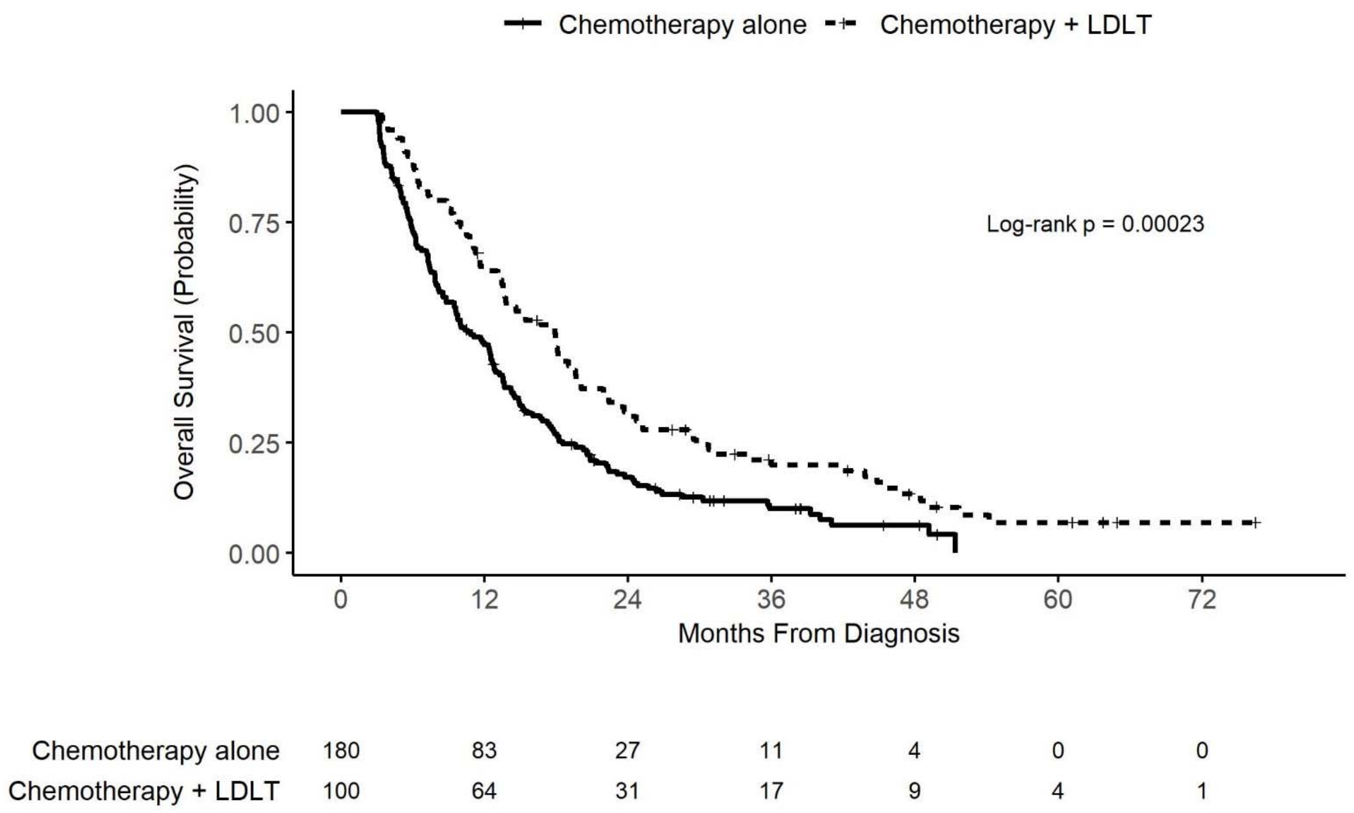

© 2019 Sebastian NT et al. *JAMA Network Open.*

(B) 6 Months

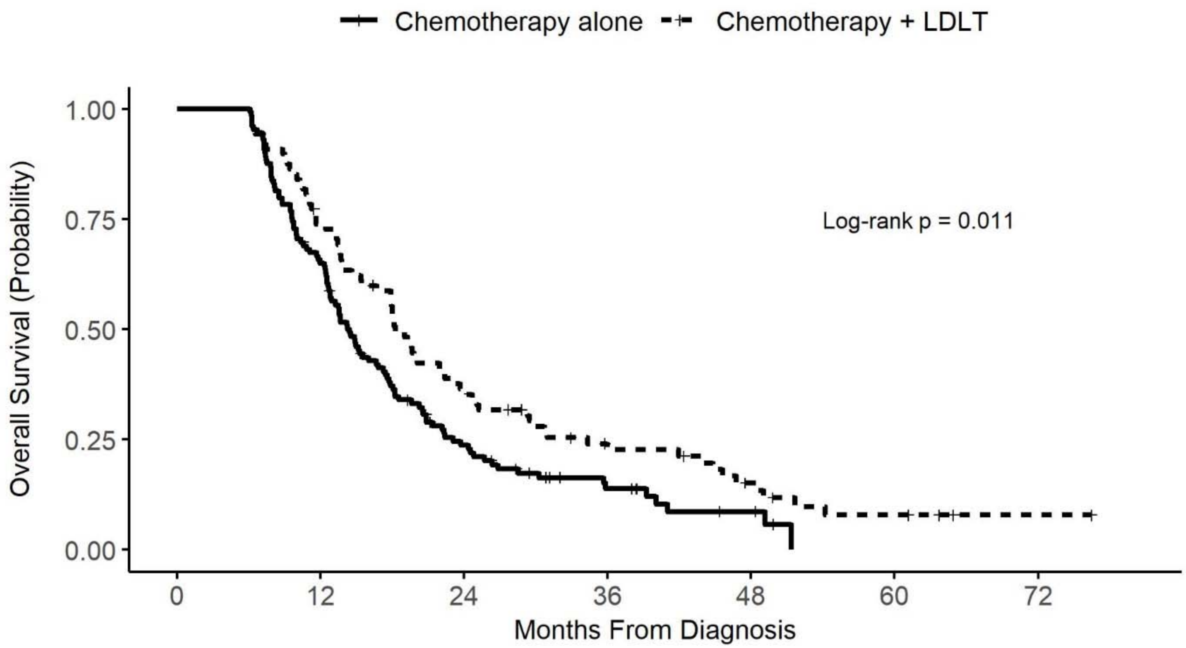

Chemotherapy alone	129	83	27	11	4	0	0
Chemotherapy + LDLT	88	64	31	17	9	4	1

© 2019 Sebastian NT et al. *JAMA Network Open.*

(C) 12 Months

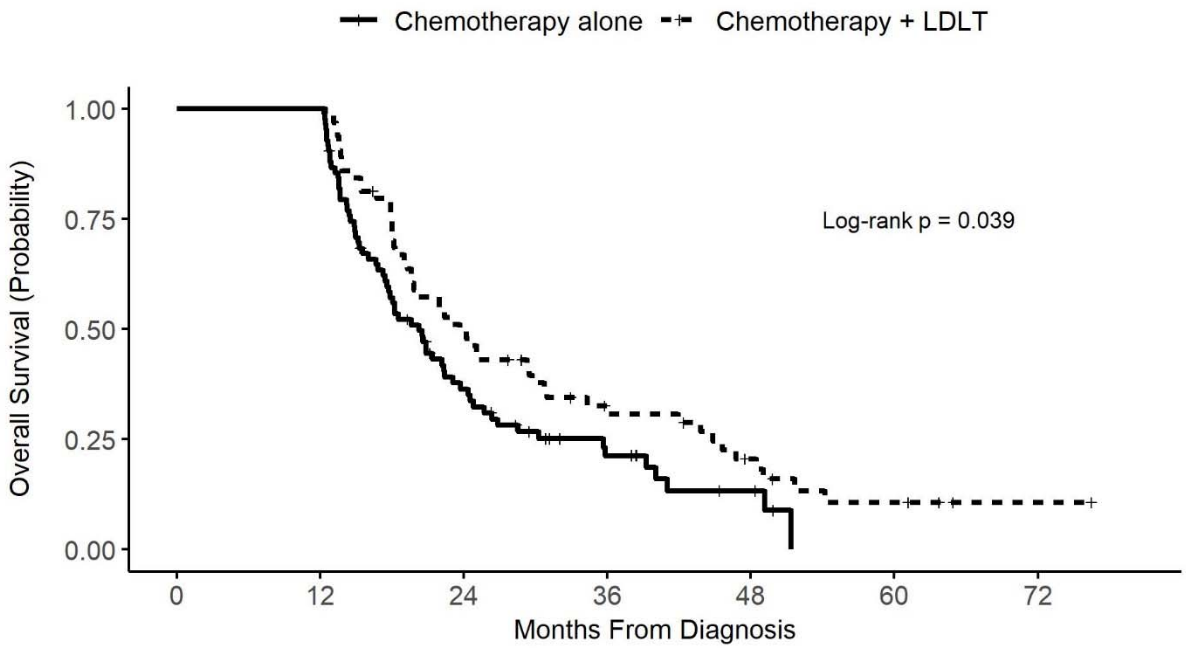

	0	12	24	36	48	60	72
Chemotherapy alone	83	83	27	11	4	0	0
Chemotherapy + LDLT	64	64	31	17	9	4	1

© 2019 Sebastian NT et al. *JAMA Network Open.*

eTable. Patient and Disease Characteristics of the Propensity Matched Cohort, Stratified by Receipt of Liver-Directed Local Therapy (LDLT)

Characteristic	Chemotherapy Alone (n = 104)	Chemotherapy + LDLT (n = 104)	P
**Age**			
Median (IQR)	62.0 (54 - 70)	61.5 (54.0 - 70.2)	0.76†
**Sex**			
Male	47 (45.2%)	46 (44.2%)	
Female	57 (54.8%)	58 (55.8%)	1.00‡
**Race**			
White	94 (90.4%)	90 (86.5%)	
Black	8 (7.7%)	9 (8.7%)	0.55‡
Other	2 (1.9%)	5 (4.8%)	
**Charlson Comorbidity Score**			
0	76 (73.1%)	73 (70.2%)	
1	21 (20.2%)	25 (24.0%)	0.69‡
2	7 (6.7%)	5 (4.8%)	
3+	0 (0.0%)	1 (1.0%)	
**Insurance Status**			
No Insurance	4 (3.8%)	5 (4.8%)	
Private	55 (52.9%)	51 (49.0%)	0.83‡
Government	45 (43.3%)	48 (46.2%)	
**Treatment Facility**			
Community Cancer Program	5 (4.8%)	5 (4.8%)	
Comprehensive Community Cancer Program	26 (25.0%)	32 (30.8%)	0.82‡
Academic/Research	62 (59.6%)	56 (53.8%)	
Integrated Network Cancer Program	11 (10.6%)	11 (10.6%)	
**Year of Diagnosis**			
Median (IQR)	2011 (2009 - 2013)	2011 (2009 - 2013)	0.49†
**T-Stage§**			
1	13 (12.5%)	15 (14.4%)	

© 2019 Sebastian NT et al. *JAMA Network Open.*

2<br>3<br>4	41 (39.4%)<br>40 (38.5%)<br>10 (9.6%)	36 (34.6%)<br>38 (36.5%)<br>15 (14.4%)	0.69‡
**Tumor Focality**<br>Unifocal<br>Multifocal	 28 (26.9%)<br>76 (73.1%)	 35 (33.7%)<br>69 (66.3%)	 0.37‡
**N-Stage§**<br>0<br>1	 45 (43.3%)<br>59 (56.7%)	 46 (44.2%)<br>58 (55.8%)	 1.00‡
**Bone Metastasis**<br>No<br>Yes	 90 (86.5%)<br>14 (13.5%)	 95 (91.3%)<br>9 (8.7%)	 0.38‡
**Lung Metastasis**<br>No<br>Yes	 90 (86.5%)<br>14 (13.5%)	 91 (87.5%)<br>13 (12.5%)	 1.00‡
**Distant Lymph Node Metastasis**<br>No<br>Yes	 59 (56.7%)<br>45 (43.3%)	 61 (58.7%)<br>43 (41.3%)	 0.89‡

Abbreviations. IQR- interquartile range.

† Mann-Whitney U (Wilcoxon rank-sum) test

‡ Fisher’s exact test

§ American Joint Committee on Cancer (AJCC) 7th edition

© 2019 Sebastian NT et al. *JAMA Network Open.*
